# Inside the Asylum
*Previous articles appeared on August 13 and August 20, p. 346.


**Published:** 1921-08-27

**Authors:** 


### THE PROBLEM OF THE INSANE.

### III.—Inside the Asylum.*

It is not possible to criticise the general dietary in asylums by taking a specific instance of the methods pursued in one institution, especially if, as in the example adduced by Dr. Lomax, war-time conditions prevail. There can, however, be little doubt that in asylums there is a rather wearisome repetition of similar dishes. It is not so much a question of materials: it is rather one of lack of imagination in the cooking. Judged by their appearance, the asylum patients do at any rate receive an adequate diet, though during the war it seems to be undoubted that they did suffer in this respect. This and the absence of a good deal of the skilled nursing which resulted from attendants being called up to serve in the Army—but particularly on account of the former factor—would account for the greatly increased death-rate which supervened. Nothing should be allowed to interfere, in normal times, with the adequacy of the diet, while every effort should be made to introduce as much variety as possible. On the other hand, it is not necessary to indulge fads and fancies, and it should be remembered that the great majority of the patients in the county asylums have been accustomed during their home-life often to very simple and more often to badly cooked food, so that they are better off in the asylum in this respect than they have been outside. Attention to diet should be particularly directed to that of the curable cases: it is they who specially require the most nutritive and most easily assimilated food.

A great point has been made of the so-called "barrack-like" appearance of the large asylums. Probably it would have been better to adopt the villa system, which is already partly in operation; but we *have* these buildings, and in these anti-waste days we must make use of them. In any case, are these spacious and airy wards and dormitories really so prejudicial to health? If so, why is there no outcry against our big general hospitals, which are certainly "barracks," and which seldom have such favourable environment as the asylums? Again, why should different kinds of surgical or of medical cases be nursed in the same wards? Either the insane must be kept in wards in association or they must be put into single rooms. But if they are associated—the acute cases, at any rate—they will have a prejudicial effect on one another; and if they are in single rooms they will shriek like lost souls to get out! It is an impasse—for the theorists. It is yet to be proved that the average case of insanity is harmed by association with other patients; while, on the other hand, it is a matter of common experience that patients who have been the centre of attention in their own family circle or in nursing homes improve rapidly when sent to an asylum. This is especially true of the querulous or hypochondriacal melancholic. When the acute symptoms begin to pass off the patient should be moved to a convalescent ward—and this is the procedure in any well-managed asylum.

In addition to food, fresh air, and rest where there is excitement, agitation, or bodily weakness, one of the most important adjuncts to treatment is occupation. In regard to this last factor the public asylums are in many ways better off than the private ones, for they have their workshops, farm, etc. On the other hand it must not be forgotten that during the acute phases of insanity patients are incapable of occupying themselves. They are too restless and cannot concentrate their attention, or they are too much influenced by delusions or hallucinations to be able to think of anything else. Naturally the nurses and attendants are only too glad to have the help of the convalescing and of the chronic patients: and in most asylums there is no difficulty in obtaining little "extras" for the workers. The critics appear to think that the attendants delight in having their patients consigned to "cells" and generally in "putting it across" them. A little consideration should lead the critics to realise that the attendants have to live with their charges, and that, therefore, it is more pleasant to be on good terms with them. Of course there are cases where attendants have mishandled patients: a glance at the record of most asylums will probably show that attendants have been summarily dismissed or even prosecuted. But these cases are rare, and no system that can be devised will eliminate this trouble until human nature alters. It is all very well to talk from a comfortable armchair about the poor lunatic and of the necessity for always feeling benevolent towards him, but let the average person be made to associate with even one troublesome and mischievous patient for several days, and then let us examine his record! Suppose the patient has spat in his nurse's face, remarking, "You mustn't touch me because I'm a lunatic!" as a preliminary to other acts of a similar character. It is not suggested that the attendants should retaliate, but let us at least be just and realise the provocation they have to experience, and which they do so well refrain from responding to.† On the positive side the amount of good they do towards the cure of patients is incalculable. It is a wise provision to see that the attendants and nurses are happy and comfortable, for such a condition of affairs inevitably reacts upon the patients to their greater well-being. Much remains to be done in this respect, and it is a pity that it should be necessary still for the staff of an asylum to demand what the prevision of the authorities should enable them to provide unasked. So long as conditions remain unsatisfactory for the staff it is no use grumbling and threatening when they form a trade union to enforce their rights.

† "A madman may be not merely a mindless animal; he may be at times more degraded than a wild beast." This is Dr. Lomax's own comment, "Experiences of an Asylum Doctor," p. 177.

* Previous articles appeared on August 13 and August 20, p. 346.

